# Characteristic and Phylogenetic Analysis of the Complete Chloroplast Genomes of Three Medicinal Plants of Schisandraceae

**DOI:** 10.1155/2020/3536761

**Published:** 2020-10-16

**Authors:** Dachuan Zhang, Jiahao Wang, Liang Xu, Yanping Xing, Tingting Zhang, Shengnan Li, Yanyun Yang, Guihua Bao, Wuliji Ao, Tingguo Kang

**Affiliations:** ^1^School of Pharmacy, Liaoning University of Traditional Chinese Medicine, Dalian 116600, China; ^2^Liaoning Quality Monitoring and Technology Service Center for Chinese Materia Medica Raw Materials, Dalian 116600, China; ^3^School of Mongol Medicine, Inner Mongolia University for Nationalities, Tongliao 028000, China

## Abstract

*Schisandra chinensis*, which has a high development value, has long been used as medicine. Its mature fruits (called Wuweizi in Chinese) have long been used in the famous traditional Chinese medicine (TCM) recorded in the “Chinese Pharmacopoeia.” Chloroplasts (CP) are the highly conserved primitive organelles in plants, which can serve as the foundation for plant classification and identification. This study introduced the structures of the CP genomes of three Schisandraceae species and analyzed their phylogenetic relationships. Comparative analyses on the three complete chloroplast genomes can provide us with useful knowledge to identify the three plants. In this study, approximately 5 g fresh leaves were harvested for chloroplast DNA isolation according to the improved extraction method. A total of three chloroplast DNAs were extracted. Afterwards, the chloroplast genomes were reconstructed using denovo combined with reference-guided assemblies. General characteristics of the chloroplast genome and genome comparison with three *Schisandraceae* species was analyzed by corresponding software. The total sizes of complete chloroplast genomes of *S. chinensis*, *S. sphenanthera*, and *Kadsura coccinea* were 146875 bp, 146842 bp, and 145399 bp, respectively. Altogether, 124 genes were annotated, including 82 protein-coding genes, 34 tRNAs, and 8 rRNAs of all 3 species. In SSR analysis, only *S. chinensis* was annotated to hexanucleotides. Moreover, comparative analysis of chloroplast *Schisandraceae* genome sequences revealed that the gene order and gene content were slightly different among *Schisandraceae* species. Finally, phylogenetic trees were reconstructed, based on the genome-wide SNPs of 38 species. The method can be used to identify and differentially analyze Schisandraceae plants and offer useful information for phylogenetics as well as further studies on traditional medicinal plants.

## 1. Introduction

Wuweizi (*Schisandra chinensis*), first recorded in “Shennong's Herbal Classic of Materia Medica,” has long been used in traditional Chinese medicine (TCM) as a top grade medicine; specifically, it has been utilized as a tonic medicine for about 2000 years [1]. In many Asian countries like Japan and South Korea, Schisandra is also listed as a pharmacopoeia variety [2, 3]. Wuweizi (*Schisandra chinensis*), Nanwuweizi (*Schisandra sphenanthera*), and Heilaohu (*Kadsura coccinea*) have displayed medicinal value. Nanwuweizi (*Schisandra sphenanthera*) is the same as Wuweizi in historical medicine; both of them were listed separately in the 2000 China Pharmacopoeia. Wuweizi is often used as a diarrhea antispasmodic agent in clinical practice, and it has astringent solidifying, Qi and fluid replenishing, as well as kidney and heart tonifying effects [1]. *K. coccinea* is known as the “magic longevity fruit,” which can be eaten as a fruit, and its root and root skin can be used for medicinal purposes, as recorded in “Gui Medicine.” Besides, it can promote qi and activate blood circulation, reduce swelling, and relieve pain [4].

Chloroplasts are important organelles for photosynthesis in plants, which have distinct physiological characteristics, such as relatively conservative and single parental inheritance [5, 6]. Chloroplasts can be used for plant species identification, hybridization, and phylogenetic analysis. In 1986, Japanese scientists had first obtained the complete chloroplast genome sequence of plants in tobacco [7]. With the rapid development of high-throughput sequencing in recent years, this emerging technology has been applied to investigation in various fields. For instance, Li et al. had investigated the structural characteristics and genetic evolution of Magnoliaceae plants through chloroplast genome-wide analysis of *Magnolia grandiflora* in 2013, and had verified the negative correlation of the IR region length in some Magnoliaceae plants with the pseudogene length *ψycf*1 [8]. In 2013, Yang et al. had carried out chloroplast genome-wide structural analysis to identify and analyze various plants of genus Cymbidium, and examined their genetic relationships [9]. Their results proved that the correlation between species based on organelle gene sequencing was reliable.

Magnoliaceae has always been a research hotspot. So far, the chloroplast genomes of about 20 plant species have been sequenced [10]. Typically, *Magnolia* is also a controversial group in botany, which is mainly attributed to the classification of botany. In the APG IV system, Schisandraceae includes *Schisandra* and *Illicium*. In 2007, Hansen et al. analyzed the chloroplast gene structures of four plants, including medicinal anise [11]. In 2017, Guo et al. examined the chloroplast genes of *S. chinensis* and determined that the *Illicium* genus was a sister branch of *Schisandra* [12]. In 2018, Li and Zheng analyzed the chloroplast genomes of *K. coccinea* and obtained the evolutionary structure of *K. coccinea* by means of phylogenetic tree analysis [13]. Modern methods have been utilized to analyze chloroplasts, such as simple sequence repeat (SSR) analysis, IR boundary analysis, and phylogenetic analysis. Of them, SSR is frequently used to identify species and analyze the genetic difference. Previous phylogenetic research focusing on the phylogenetic tree of consensus protein clustering has only taken into consideration the coding region variation, which has certain limitations. Therefore, this paper had been carried out with the aim of analyzing the phylogenetic tree of genome-wide single-nucleotide polymorphisms (SNPs), with the consideration of variations in the coding region and the noncoding region. Notably, our analysis was more comprehensive, and our results were more accurate and reliable.

In the 2015 China Pharmacopoeia, all the three medicinal plants examined in this study are classified into Magnoliaceae; however, in the latest APG IV classification system [14], Schisandraceae is classified into a separate family, which includes *Illicium* L., *Kadsura* Kaempf., and *Schisandra* Michx. Liu and Hu suggested that Schisandraceae be considered as one family, and divided and categorized as the *Illicium* genus through comparing plant morphology, palynology, and cytology [15–18]. It is believed that the *Illicium* genus is a model genus, which is also known as *Illicium* L., as suggested by Zhang. From the perspective of chloroplast gene organization of Magnoliaceae, Schisandraceae, and star anise, this paper had provided the novel foundation for plant classification [19]. There are currently about 80 species of Schisandraceae, including 34 medicinal plants [20, 21]. The commonly used agents in Schisandraceae are found in *S. chinensis* and *S. sphenanthera*, and many methods can be used for identification, like chemistry and molecular pharmacy [22–24].

Various plants of Schisandraceae have exhibited high economic and medicinal value. This paper is aimed at analyzing the complete chloroplast genomes of *S. chinensis*, *S. sphenanthera*, and *K. coccinea*, so as to explore the basis for identification and genetic relationships among these three. Notably, *S. chinensis* and *S. sphenanthera* have not been accurately distinguished in the history of medication use, and mixed phenomena can be observed. Therefore, exploring the differences at the gene level between these two is not only conducive to identifying these two traditional Chinese medicines, but it also lays the vital genetic foundation for the future cultivation of *S. chinensis* and *S. sphenanthera*. This study is aimed at exploring the intrinsic relationship and difference in the chloroplast gene structure of *S. chinensis* through internal analysis of *S. chinensis* and *S. sphenanthera*, as well as *K. coccinea*. Besides, phylogenetic analysis of Schisandraceae with other families was also carried out to obtain the position of Schisandra during phylogenetic evolution.

## 2. Materials and Methods

### 2.1. Plant Materials

The fresh *S. chinensis* leaves were collected from Dalian, China (E121°52′34.96^″^, N39°03′43.96^″^). The fresh leaves of *S. sphenanthera* were collected from Shangluo, China (E110°02′27.55^″^, N33°55′34.10^″^). The fresh leaves of *K. coccinea* were obtained from the channel Xufeng Chinese Herbal Medicine Cooperative (Huaihua, Hunan Province, China (E110°05′03.05^″^, N27°28′30.10^″^). These three species were identified by Dr. Tingguo Kang from the Liaoning University of Traditional Chinese Medicine in Shenyang, China. Permission for using plant fruit samples in biological experiments had been granted. Plant samples were deposited in the herbarium of Liaoning University of Traditional Chinese Medicine and the genomic DNA was stored in the Key Laboratory of Traditional Chinese Medicine in the University (Dalian, China, 116600).

### 2.2. Chloroplast DNA Extraction and Sequencing

Approximately 5 g fresh leaves were harvested for chloroplast DNA isolation according to an improved extraction method [25]. After DNA isolation, 1 *μ*g purified DNA was fragmented, which was then utilized to construct the short-insert libraries (insert size of 430 bp) in accordance with the manufacturer's protocol (Illumina), followed by sequencing on the Illumina HiSeq 4000 [26].

Raw reads were filtered prior to assembly, so as to remove reads with adaptors, or those with a quality score of <20 (*Q* < 20), or those containing ≥10% uncalled based (“N” characters) and duplicated sequences. Afterwards, the chloroplast genomes were reconstructed using denovo combined with reference-guided assemblies, and the following three steps were adopted for chloroplast genome assembly [27]. First of all, the filtered reads were assembled into contigs using SOAP denovo 2.04 [28]. Secondly, the assembled contigs were aligned to the reference genomes of two species using BLAST, and then the aligned contigs (≥80% similarity and query coverage) were ordered according to the reference genomes. Thirdly, clean reads were mapped to the assembled draft chloroplast genomes, so as to correct the wrong bases, and later the majority of gaps were filled by means of local assembly.

### 2.3. Genome Assembly and Annotation

The chloroplast genes were annotated by the online DOGMA tool, and default parameters were used to predict protein-coding genes, and to transfer RNA (*trn*A) genes as well as ribosome RNA (rRNA) genes. Subsequently, a comprehensive chloroplast genome-wide Blast search was performed among 5 databases [29], namely, the Kyoto Encyclopedia of Genes and Genomes (KEGG), the Clusters of Orthologous Groups (COG), the Nonredundant Protein Database (NR), Swiss-Prot, and Gene Ontology (GO) [30–37]. At the same time, the SSR software MIcroSAtellite (MISA) (http://pgrc.ipk-gatersleben.de/misa/) was employed to identify the SSR sequences, and tandem repeats of 1–6 nucleotides were considered as microsatellites. Moreover, MISA (MIcroSAtellite; http://pgrc.ipk-gatersleben.de/misa) was utilized to detect the genomes of SSR in the chloroplast, and the parameters were set as follows: >11 for mononucleotides, >6 for dinucleotides, >5 for trinucleotides, >5 for tetranucleotides, >5 for pentanucleotides, and >5 for hexanucleotides. In addition, the maximal number of bases interrupting 2 SSRs in a compound microsatellite was set as 100. This paper had focused on the perfect repeat sequences. The mVISTA was used for similarity analysis between these five Magnoliaceae species.

### 2.4. Chloroplast Genome Mapping

The chloroplast genomes of *S. chinensis*, *S. sphenanthera*, and *K. coccinea* were exported in the GenBank format using the Sequin software, and then mapped based on the annotation results (http://ogdraw.mpimp-golm.mpg.de/index.shtml). Finally, their complete chloroplast genomes were submitted to the NCBI GenBank database.

### 2.5. Phylogenetic Analysis

The genome sequences of 38 species were utilized to analyze the phylogenetic relationships on the basis of genome-wide SNPs of the 38 species. Additionally, the Maximum Likelihood (ML) phylogenetic tree was constructed using MEGA 6.0.

## 3. Results and Discussion

### 3.1. General Characteristics

The chloroplast genes of three medicinal plants were detected (Table 1). Results suggested that the full-length chloroplast genes of *S. chinensis*, *S. sphenanthera*, and *K. coccinea* were 146875 bp, 146842 bp, and 145399 bp, respectively, while the GenBank numbers were SRX4282568, SRX4282569, and SRX4282570, respectively, which were similar to the chloroplast genome sizes of other Schisandraceae plants. These results had indicated the relatively conservative evolution of chloroplast genes in Schisandraceae plants. Moreover, the GC contents of *S. chinensis*, *S. sphenanthera*, and *K. coccinea* were 43.11%, 39.60%, and 39.70%, respectively. The LSC lengths were 96686 bp, 95627 bp, and 94287 bp, separately; the SSC lengths were 18270 bp, 18280 bp, and 18039 bp, respectively; and the IR (IRa, IRb) lengths were 15958 bp, 16466 bp, and 16535 bp, separately. Moreover, it was known from the sequencing results that LSC, SSC, and IRa (IRb) had close lengths in the four regions of chloroplast genes, and the numbers of total genes, encoded genes, rRNA genes, and *trn*A genes were highly consistent among them. Besides, sex indicated quite close kinship of these three chloroplast genes.

**Figure 1:**
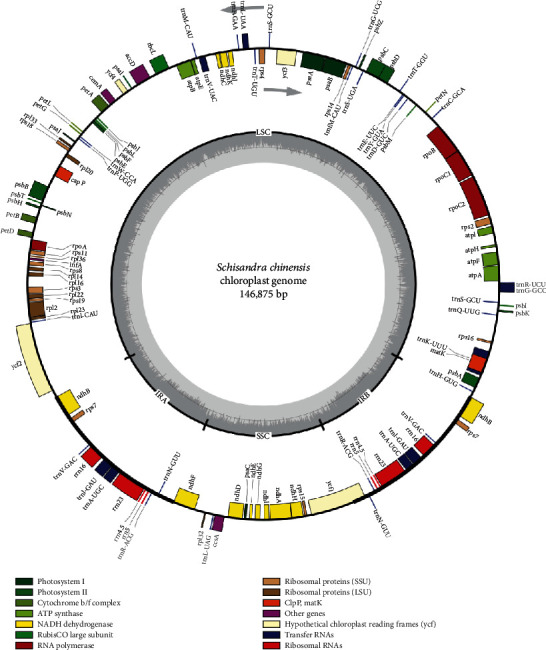
Chloroplast genes map of *S. chinensis.*

The annotated genes of *S. chinensis*, *S. sphenanthera*, and *K. coccinea* were generally identical, as presented in Figure 1 (the maps of *S. sphenanthera* and *K. coccinea* are shown in Figures S1 and S2), but certain differences were also observed (Table 2). For example, *S. sphenanthera* had an *rps*12 sequence in both IRa and IRb, which was not seen in *S. chinensis*. In addition, the *clp*P sequences of *rps*16, petB, and *rpl*16 in the LSC region of *S. chinensis* were 218 bp, 641 bp, 410 bp, and 1160 bp in length, which were markedly shorter than the 1079 bp, 1408 bp, 1355 bp, and 1901 bp in *S. sphenanthera*. Additionally, the *rpl*16 sequence of *S. sphenanthera* contained two exons and one exon, which were not seen in *S. chinensis*; thus, the base species of *S. chinensis* was identified based on these differences. Compared with *S. chinensis*, *K. coccinea* had an additional *rps*12 sequence in the IRa and IRb regions, as well as a D2 type *ndh*B in the IRb region. Besides, the lengths of *rps*16, *rpl*16, *pet*B, *pet*D, and *clp*P of *S. chinensis* were 218 bp, 410 bp, 641 bp, 533 bp, and 1160 bp, respectively; while those of *K. coccinea* (1085 bp, 1371 bp, 1408 bp, 1254 bp, and 1978 bp) were longer than those of *S. chinensis*. When comparing *S. sphenanthera* with *K. coccinea*, the *pet*D in the LSC region of *S. sphenanthera* was shorter, the detailed data were the same as those presented above, and the IRb region of *K. coccinea* was D2 type *ndh*B. The high gene similarity was ascribed to the species similarity, which also indicated the relatively conservative family evolution of *S. chinensis*.

An intron plays a vital part in regulating gene expression. Recent studies suggest that many introns can increase the expression and timing of foreign genes at specific locations, which can partially determine the plant-specific traits. Therefore, introns can serve as a useful approach to improve the required agronomic traits [38]. There were 12 intron-coding genes in the *S. chinensis* chloroplast DNA, of which *ycf3*, *rps12*, *rps12-D2*, and *clpP* contained two introns, whereas the remaining eight genes had only one intron (Table 3). By contrast, there were 14 genes containing introns in the *S. sphenanthera* chloroplast DNA, among which, *ycf3*, *rps12*, *rps12-D2*, and *clpP* contained two introns, while the other eight were the same as those of *S. chinensis*. However, *S. sphenanthera* had two more exon genes than *S. chinensis*, which were *petD* and *rpl16*; therefore, they could be used to identify north *S. chinensis* species from the south *S. chinensis* species. Meanwhile, the number and species of *K. coccinea* introns were the same as those of *S. chinensis*. The overall comparison showed that the exons contained in these three medicinal plants were basically the same in size, and the intron size was very small, with only a few bp between them, suggesting the close kinship among these three plants. Investigating the introns among these three plants contributed to improving plant resilience and developing new varieties. In addition, there was a high degree of similarity among the three, which could be used for reference in plant breeding of the latter three.

### 3.2. Repeat Structure and Simple Sequence Repeat Sequence Analysis

Many repeats are present in gene deserts, although whole-genome sequencing has shown that they can occur in functional regions as well. Repeats of more than 30 bases were considered as the long repeats.

**Figure 2:**
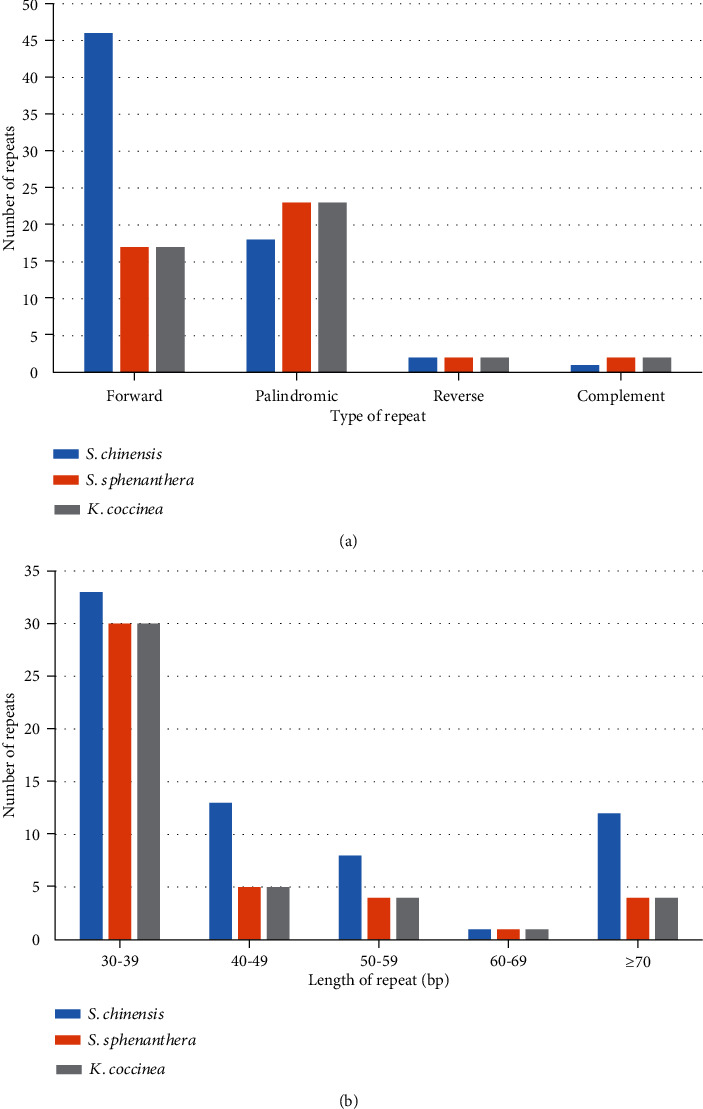
Analysis of repeated sequences in the 3 cp genomes.

A total of 67, 44, and 44 pairs of repeats were identified in the *S. chinensis*, *S. sphenanthera*, and *K. coccinea* cp genomes (Figure 2(a)). *S. chinensis* contained 46 forward repeats, 18 palindromic repeats, 2 reverse repeats, and 1 complement repeat. *S. sphenanthera* and *K. coccinea* contained 17, 23, 2, and 2 repeats, respectively, with repeat lengths ranging from 30 to 131 bp (Figure 2(b)).

A Simple Sequence Repeat (SSR) is a PCR-based highly efficient molecular labeling technique. The SSRs are constantly found to be highly polymorphic, easily visible, stable, and codominant, whereas the structures of chloroplast genomes are simple and relatively conservative. Single parental inheritance, together with other characteristics, has been extensively used in species identification and genetic diversity analysis. Because of the characteristics of neutral markers, the highly variable numbers of repeats, and the relative conservatism of flanking sequences of SSRs, they are widely distributed in the genome of organisms. Microsatellites or SSRs play a major role in polymorphism analysis and in marker-assisted selection [39].

**Figure 3:**
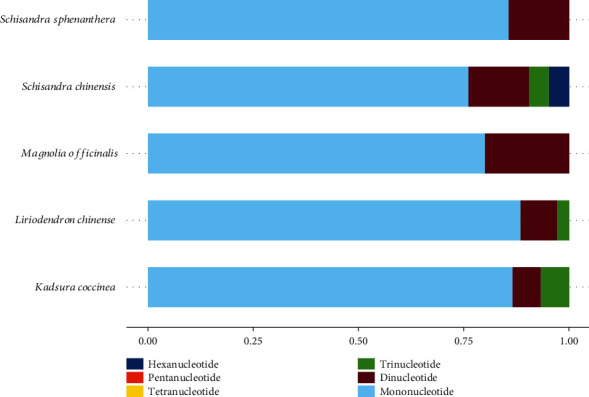
SSR abundance map of 5 species plants.

A total of 57 eligible SSR loci were detected in the genomes of three chloroplasts of the *S. chinensis* family (Table 4, Figure 3). Of them, *S. chinensis* contained 16 single-nucleotide, 3 dinucleotide, 1 trinucleotide, and 1 hexanucleotide repeat gene sequences. *S. sphenanthera* contained 18 single-nucleotide and 3 dinucleotide repeat gene sequences. *K. coccinea* had 13 single-nucleotide, 1 dinucleotide, and 1 trinucleotide repeat gene sequences. Obviously, there was distinct difference between these three plants. Compared with the two Magnoliaceae plants, the three Schisandraceae plants had closer SSR types and abundance.

Gene sequence abundance is inversely related to the length of the repeat gene sequence. Among all the SSRs of these three plants, A/T was the most frequently repeated sequences, followed by AT/AT, and they had accounted for 94.7% of the total sequences. There was a certain base preference regarding the base composition of a single-nucleotide to a trinucleotide repeat sequence, suggesting that the Schisandraceae plant was mainly composed of the A/T-rich repeat sequence. The single nucleotides were identical, and the SSR loci of other Schisandraceae species were searched by the same parameters and their repeat sequence types as well as abundance percentages were compared. Our results suggested that the types and abundance of repeat sequences were conservative between Schisandraceae species, which had laid a certain foundation for further searching for the universal SSR markers between the Schisandraceae species.

### 3.3. Comparative Analysis of the IR Boundary Regions

**Figure 4:**
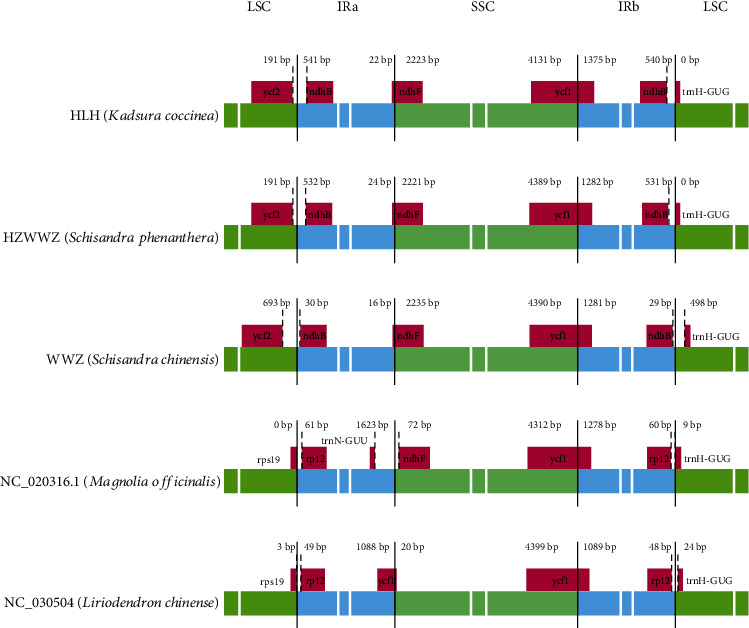
Comparisons of LSC, SSC, and IR boundary regions among three Schisandraceae species and two Magnoliaceae species.

The size of chloroplast genomes mainly depends on the contraction and expansion of the IR region [40]. The three medicinal plants of Schisandraceae were internally compared, and the comparison between Schisandraceae and Magnoliaceae families showed that plants within the same family were more similar (Figure 4). *S. sphenanthera* showed a higher similarity to *S. chinensis*, the length between LSC and IRa was the same, the gene length between *ycf1* and *ndh*b was 723 bp, and the slightly larger *K. coccinea* was 732 bp. However, the distance between the *ycf1* and *ndhB* genes of *S. chinensis* and *S. sphenanthera* differed greatly from the boundary. The boundary distance between *ycf*1 and the IR region was also different. *K. coccinea* was 191 bp, and it was identical to that of *S. sphenanthera*, while *S. chinensis* was 693 bp, which was significantly larger than the former two. Such difference could also be detected between LSC and IRb, as well as the distance between the *ndhB* gene of *S. sphenanthera* and *K. coccinea*, with the boundary distances of 531 bp and 540 bp, respectively, which were evidently larger than the 29 bp of *S. chinensis*. The distance from the *trnh*-*GUG* gene of *S. chinensis* to the IRb region was greater than those of *S. sphenanthera* and *K. coccinea*. In summary, the three medicinal plants of Schisandraceae could be distinguished based on the IR region; besides, distance analysis between the IR region and the gene also revealed a closer relationship between *S. chinensis* and *S. sphenanthera*. Moreover, the relative analysis of the two Schisandraceae and Magnoliaceae plants showed higher similarity between *Magnolia* and *Liriodendron*, and a greater difference between Schisandraceae, which accounted for the correct division of Schisandraceae into one family.

### 3.4. Phylogenetic Analysis

**Figure 5:**
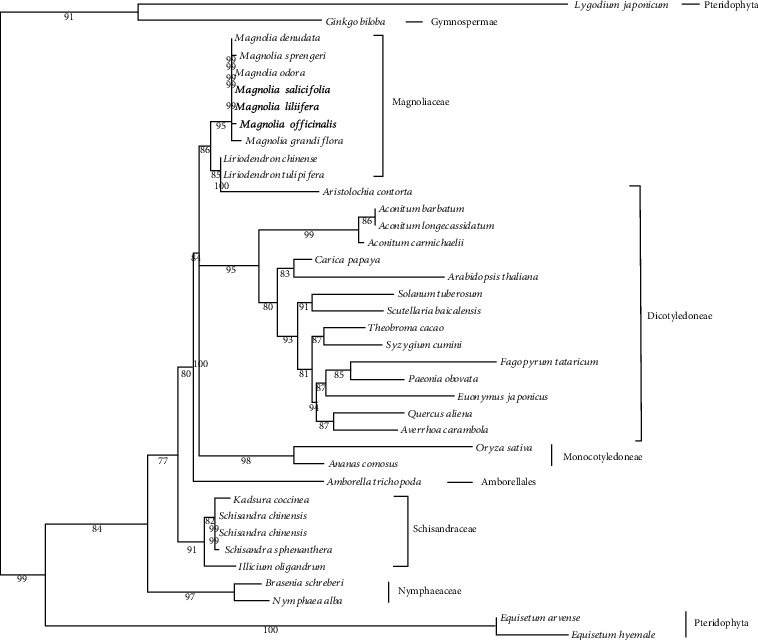
Molecular phylogenetic tree of genome-wide SNPs from 38 species (1000 bootstrap replicates).

In this paper, the ML (map) and phylogenetical tree were constructed (Figure 5) based on the genome-wide SNPs of 38 species; 25 of the 36 nodes in the ML tree had ≥90% of the support values, among which, 3 were 100% and only one was <80%. Using the APG IV plant classification system, Schisandraceae was assigned to the *Malva sylvestris* located in the base group of angiosperms, and consistent results with the latest classification system were obtained. It was observed from Table 4 that Schisandraceae was listed as a separate item. The three Schisandraceae plants had quite close relationships, among which, *S. chinensis* was closer to *S. sphenanthera* than *K. coccinea*, which was consistent with our real-time taxonomy. It was worth mentioning that, in the ML tree, *Magnolia officinalis* and *Liriodendron chinense*, together with *S. chinensis*, were divided into two, which also showed that it was reasonable to modernize *S. chinensis* from Magnoliaceae and separate it into one family. The *Illicium* and Schisandraceae had a similar relationship, which was far from the *M*. *officinalis* and *L*. *chinense* of Magnoliaceae. Hopefully, such data will provide certain help in the subsequent botanical classification of these plants.

The MP tree had indicated the same result as those of the ML tree. Specifically, the three plants and the *Illicium* genus in Schisandraceae were divided into one, and *M*. *officinalis* and *L*. *chinense* were also divided into one, indicating that the Schisandraceae and the octagonal fennel were the sister branches that had closer kinship. *L. chinense* was closer to *M*. *officinalis*, which was consistent with our results based on the APG IV classification system, indicating that the chloroplast genomes could accurately identify the genetic relationship between different species.

### 3.5. Genome Comparison Analysis

**Figure 6:**
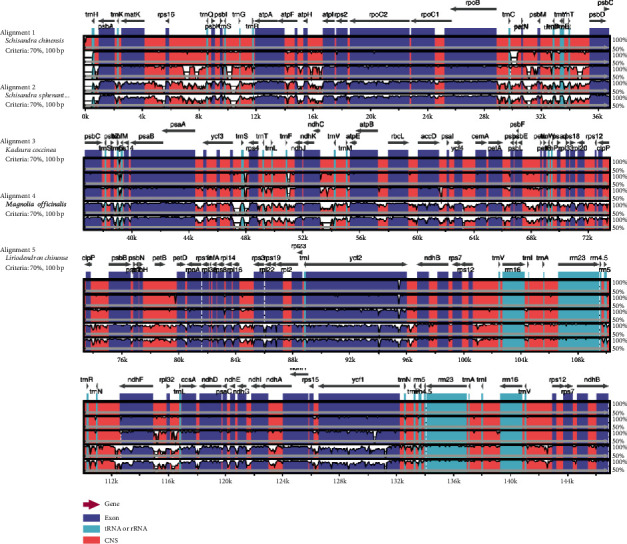
Comparison of chloroplast genomes from five plants species using mVISTA.

Moreover, the genomic structures in the genera of Schisandraceae were compared using the mVISTA software in the Shuffle-LAGAN mode, with the genome-wide *S. chinensis* chloroplast being used as the reference (Figure 6). The complete genomes of 5 plant species are used for comparison. Genic regions are identified using the DOGMA program, and a comparative map is prepared using mVISTA. The blue block indicates the conserved gene, the sky-blue block stands for trnA and rRNA, and the red block represents the intergenic region. Meanwhile, the white peaks indicate the regions with sequence variation among the five species. Our results suggested that the noncoding region had a higher degree of variation than the coding region, which might be ascribed to the replication correction of the IR region; as a result, genes in the IR region were more conserved than those in the LSC and SSC regions [40]. Comparison of genomes showed that the genes of *S. sphenanthera* and *S. chinensis* displayed higher degrees of similarity, followed by *K. coccinea*, *M. officinalis*, and *L. chinense*, respectively. Clearly, the genotypes of three Schisandraceae plants were more similar, and the *M. officinalis* of Magnoliaceae was more similar to *L. chinense*. This also provided evidence for the separation of Schisandraceae from Magnoliaceae.

## 4. Conclusions

In this study, the genome-wide chloroplast of three Schisandraceae plants is sequenced, and the genomes are annotated and analyzed. The molecular data identified between these three species are obtained through the comparative analysis of three medicinal plants, and the SSR comparison is the same. Under the analysis conditions, the nucleotide types and numbers of *S. chinensis* and *S. sphenanthera* are more similar, while those of *K. coccinea* are only slightly similar. These three species can be clearly identified through the boundary analysis of the IR region in three Schisandraceae plants. The phylogenetic location of Schisandraceae can be obtained through phylogenetic analysis of three Schisandraceae plants. These results prove that three *S. chinensis* plants can be identified through genome-wide analysis of chloroplasts, and the phylogenetic relationships among species can also be acquired through chloroplast-to-species evolution and genetic relationship.

## Figures and Tables

**Table 1:** Comparison of three chloroplast gene data.

Species	*S. chinensis*	*S. sphenanthera*	*K. coccinea*
Gene length (bp)	146875	146842	145399
GC (%)	43.11	39.60	39.70
LSC (bp)	96686	95627	94287
SSC (bp)	18270	18280	18039
IRa (bp)	15958	16466	16535
IRb (bp)	15958	16466	16535
Gene number	124	124	124
Protein-coding gene number	82	82	82
rRNA gene number	8	8	8
*trn*A gene number	34	34	34

**Table 2:** Lists of genomic genes of *S. chinensis*, *S. sphenanthera*, and *K. coccinea.*

Genes	*S. chinensis*	*S. sphenanthera*	*K. coccinea*
Group of genes	Gene name	Gene name	Gene name
Ribosomal RNAs	rRNA23,16^d^, 5, 4.5	rRNA23, 16, 5, 4.5	rRNA23, 16, 5, 4.5
Transfer RNAs	trnH-GUG, trnK-UUU^d^, trnQ-UUG, trnS-GCU, trnG-GCC, trnG-GCC^d^, trnR-UCU, trnC-GCA, trnD-GUC, trnY-GUA, trnE-UUC, trnT-GGU, trnS-UGA, trnG-UCC, trnM-CAU, trnS-GCU, trnT-UGU, trnL-UAA^d^, trnF-GAA, trnV-UAC^d^, trnM-CAU, trnW-CCA, trnP-UGG, trnI-CAU, trnV-GAC^d^, trnM-CAU, trnW-CCA, trnP-UGG, trnI-CAU, trnV-GAC, trnI-GAU^d^, trnA-UGC^d^, trnR-ACG, trnN-GUU, trnL-UAG, trnN-GUU, trnR-ACG, trnA-UGC^d^, trnI-GAU^d^, trnV-GAC	trnH-GUG, trnK-UUU^d^, trnQ-UUG, trnS-GCU, trnG-GCC, trnG-GCC^d^, trnR-UCU, trnC-GCA, trnD-GUC, trnY-GUA, trnE-UUC, trnT-GGU, trnS-UGA, trnG-UCC, trnM-CAU, trnS-GCU, trnT-UGU, trnL-UAA^d^, trnF-GAA, trnV-UAC^d^, trnM-CAU, trnW-CCA, trnP-UGG, trnI-CAU, trnV-GAC^d^, trnM-CAU, trnW-CCA, trnP-UGG, trnI-CAU, trnV-GAC, trnI-GAU^d^, trnA-UGC^d^, trnR-ACG, trnN-GUU, trnL-UAG, trnN-GUU, trnR-ACG, trnA-UGC2, trnI-GAU^d^, trnV-GAC	trnH-GUG, trnK-UUU^d^, trnQ-UUG, trnS-GCU, trnG-GCC, trnG-GCC^d^, trnR-UCU, trnC-GCA, trnD-GUC, trnY-GUA, trnE-UUC, trnT-GGU, trnS-UGA, trnG-UCC, trnM-CAU, trnS-GCU, trnT-UGU, trnL-UAA^d^, trnF-GAA, trnV-UAC^d^, trnM-CAU, trnW-CCA, trnP-UGG, trnI-CAU, trnV-GAC^d^, trnM-CAU, trnW-CCA, trnP-UGG, trnI-CAU, trnV-GAC, trnI-GAU^d^, trnA-UGC^d^, trnR-ACG, trnN-GUU, trnL-UAG, trnN-GUU, trnR-ACG, trnA-UGC^d^, trnI-GAU^d^, trnV-GAC
Proteins of small ribosomal subunits	rps2, 3, 4, 7^d^, 8, 11, 12^d^, 14, 15, 16, 18, 19,	rps2, 3, 4, 7^d^, 8, 11, 12^d^, 14, 15, 16, 18, 19,	rps2, 3, 4, 7^d^, 8, 11, 12^d^, 14, 15, 16, 18, 19,
Proteins of large ribosomal subunits	rpl14, 16, 2, 20, 22, 23, 32, 33, 36	rpl14, 16, 2, 20, 22, 23, 32, 33, 36	rpl14, 16, 2, 20, 22, 23, 32, 33, 36
Subunits of RNA polymerase	rpoA, B, C1, C2	rpoA, B, C1, C2	rpoA, B, C1, C2
Subunits of NADH dehydrogenase	ndhA, B^d^, C, D, E, F, G, H, I, J, K	ndhA, B^d^, C, D, E, F, G, H, I, J, K	ndhA, B^d^, C, D, E, F, G, H, I, J, K
Subunits of photosystem I	psaA, B, C, I, J	psaA, B, C, I, J	psaA, B, C, I, J
Subunits of photosystem II	PsbA, B, C, E, D, F, H, I, J, K, L, N, T, Z	PsbA, B, C, E, D, F, H, I, J, K, N, T, Z	PsbA, B, C, E, D, F, H, I, J, K, N, T, Z
Large subunit of Rubisco	rbcL	rbcL	rbcL
Subunits of cytochrome b/f complex	petA, B, D, G	petA, B, D, G	petA, B, D, G
Subunits of ATP synthase	atpA, B, E, F, H, I	atpA, B, E, F, H, I	atpA, B, E, F, H, I
Acetyl-CoA carboxylase	accD	accD	accD
C-type cytochrome synthesis gene	ccsA	ccsA	ccsA
Maturase	matK	matK	matK
Protease	clpP^d^	clpP^d^	clpP^d^
Envelope membrane protein	cemA	cemA	cemA
Conserved hypothetical chloroplast reading frames	ycf1, 2, 3, 4	ycf1, 2, 3, 4	ycf1, 2, 3, 4
Translational initiation factor	infA	infA	infA

*d*: indicates that there is a double-segment gene.

**Table 3:** Characteristics and sizes of intron and exon genes of *S. chinensis*, *S. sphenanthera*, and *K. coccinea.*

	Gene	Exon I (bp)	Intron I (bp)	Exon II (bp)	Intron II (bp)	Exon III (bp)
*S. chinensis*	rps16	220	821	39		
	rpoC1	1635	685	454		
	atpF	411	773	142		
	ycf3	140	769	220	739	123
	rps12	110	27234	25	537	231
	rps12-D2	110	70401	231	537	25
	clpP	248	559	291	799	70
	PetB	5	762	641		
	rpl2	442	652	390		
	ndhA	517	1080	561		
	ndhB	727	676	777		
	ndhB-D2	777	676	727		
*S. sphenanthera*	rps16	220	820	39		
	rpoC1	1635	685	454		
	atpF	411	767	142		
	ycf3	140	769	220	740	123
	rps12	113	27147	25	537	231
	rps12-D2	113	70336	231	537	25
	clpP	248	640	291	802	70
	PetB	5	765	647		
	PetD	7	691	525		
	rpl2	442	651	390		
	rpl16	401	946	8		
	ndhA	517	1080	561		
	ndhB	777	676	727		
	ndhB-D2	727	676	777		
*K. coccinea*	rps16	220	826	39		
	rpoC1	1635	686	454		
	atpF	411	741	142		
	ycf3	140	768	220	735	123
	rps12	113	27213	25	537	231
	rps12-D2	113	70281	231	537	25
	clpP	248	566	291	703	70
	PetB	5	762	641		
	rpl2	442	653	390		
	ndhA	517	1080	561		
	ndhB	777	676	727		
	ndhB-D2	727	676	777		

**Table 4:** Comparison of the SSR data of five chloroplast genes.

Species	*S. chinensis*	*S. sphenanthera*	*K. coccinea*	*Magnolia officinalis*	*Liriodendron chinense*
Mononucleotide	16	18	13	12	31
Dinucleotide	3	3	1	3	3
Trinucleotide	1	0	1	0	1
Hexanucleotide	1	0	0	0	0

## Data Availability

The assembled complete genome sequences of the three species were submitted to NCBI with the accession numbers SRX4282568 (*S. chinensis*), SRX4282569 (*S. sphenanthera*), and SRX4282570 (*K. coccinea*). Users could download the data as a reference for research purposes only.

## Supplementary Materials

Supplementary Materials Figure S1: chloroplast gene map of *S. sphenanthera.* Figure S2: chloroplast gene map of *Kadsura coccinea.* Table S3: long repeat sequences in *S. chinensis* chloroplast genome. Table S4: long repeat sequences in *S. sphenanthera* chloroplast genome. Table S5: long repeat sequences in *K. coccinea* chloroplast genome.
