# Supplementary material for: Characteristic and Phylogenetic Analysis of the Complete Chloroplast Genomes of Three Medicinal Plants of Schisandraceae
Source: Biomed Res Int. 2020 Oct 16;2020:3536761. doi: 10.1155/2020/3536761 (PMC7586179; doi:10.1155/2020/3536761)
Supplement: Supplementary Materials — Figure S1: chloroplast gene map of S. sphenanthera. Figure S2: chloroplast gene map of Kadsura coccinea. Table S3: long repeat sequences in S. chinensis chloroplast genome. Table S4: long repeat sequences in S. sphenanthera chloroplast genome. Table S5: long repeat sequences in K. coccinea chloroplast genome. [file 3536761.f1.docx]

**SupplementaryMaterials
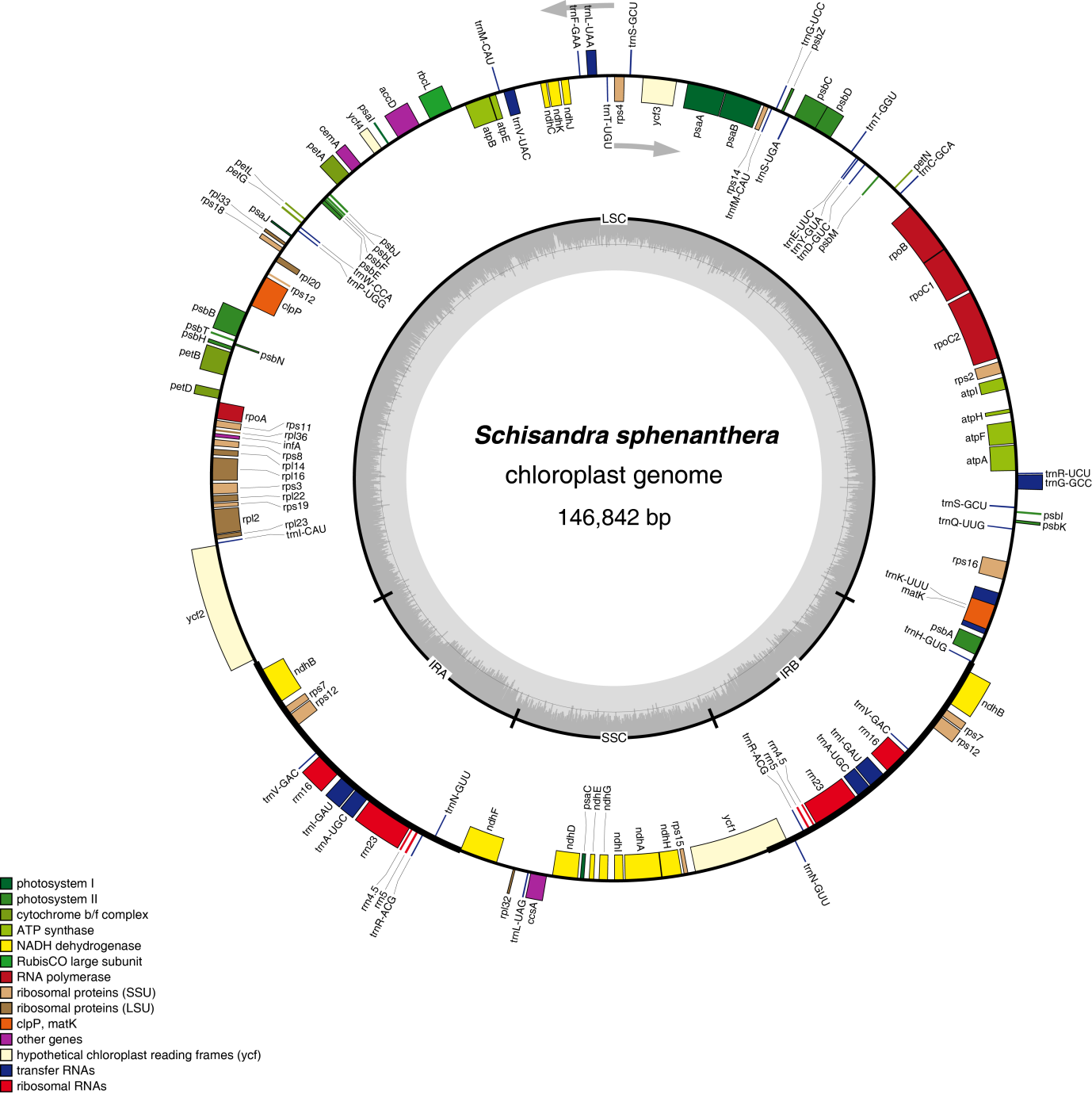
**

**Figure S1: Chloroplast genes map of *S. sphenanthera***


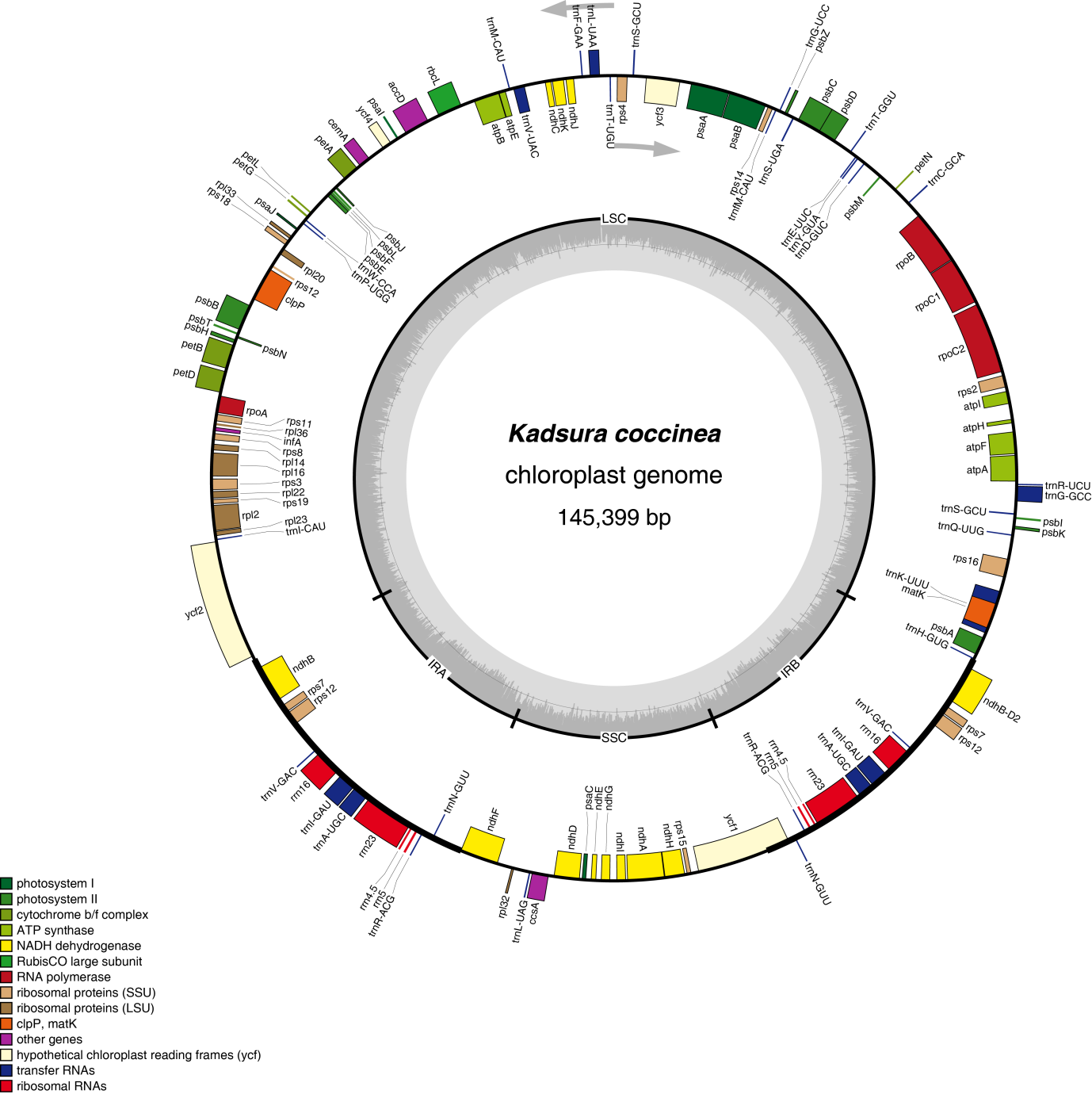


**Figure S2: Chloroplast genes map of *Kadsura coccinea***

**Table S3: Long repeat sequences in *S. chinensis* chloroplast genome**

| ID | 1Size (bp) | Type | Repeat Start 1 | Repeat Start 2 | Mismatch (bp) | E-value | Gene | Region |
| --- | --- | --- | --- | --- | --- | --- | --- | --- |
| R1 | 30 | P | 5217 | 14174 | 2 | 2.64E-04 | IGS | LSC |
| R2 | 53 | P | 8042 | 8042 | 1 | 4.72E-20 | IGS | LSC |
| R3 | 35 | P | 9748 | 47773 | 3 | 2.96E-05 | trnS-GCU、trnS-GCU | LSC |
| R4 | 31 | F | 9755 | 38089 | 3 | 5.21E-03 | trnS-GCU、trnS-UGA | LSC |
| R5 | 32 | F | 11677 | 39000 | 3 | 1.44E-03 | trnG-GCC、trnG-UCC | LSC |
| R6 | 31 | P | 16108 | 16119 | 2 | 7.05E-05 | IGS | LSC |
| R7 | 48 | P | 29134 | 29134 | 0 | 7.66E-20 | IGS | LSC |
| R8 | 30 | P | 29328 | 126401 | 3 | 5.21E-03 | IGS | LSC、SSC |
| R9 | 34 | P | 31504 | 31504 | 0 | 2.06E-11 | IGS | LSC |
| R10 | 30 | F | 32697 | 32715 | 3 | 1.88E-02 | IGS | LSC |
| R11 | 32 | F | 32702 | 32711 | 0 | 3.29E-10 | IGS | LSC |
| R12 | 37 | P | 34956 | 34956 | 2 | 2.46E-08 | IGS | LSC |
| R13 | 30 | P | 38087 | 47773 | 3 | 1.88E-02 | trnS-UGA、trnS-GCU | LSC |
| R14 | 32 | P | 38283 | 38283 | 2 | 1.88E-05 | IGS | LSC |
| R15 | 40 | F | 39037 | 39074 | 0 | 5.02E-15 | IGS | LSC |
| R16 | 41 | F | 41275 | 43499 | 3 | 1.18E-08 | psaB、psaA | LSC |
| R17 | 31 | F | 41285 | 43509 | 2 | 7.05E-05 | psaB、psaA | LSC |
| R18 | 41 | F | 46203 | 100632 | 2 | 1.19E-10 | IGS | LSC、IRa |
| R19 | 41 | P | 46203 | 142890 | 2 | 1.19E-10 | IGS | LSC、IRb |
| R20 | 30 | F | 47875 | 47988 | 3 | 1.88E-02 | IGS | LSC |
| R21 | 31 | F | 47878 | 47986 | 1 | 4.86E-07 | IGS | LSC |
| R22 | 101 | F | 47880 | 47916 | 0 | 9.44E-52 | IGS | LSC |
| R23 | 107 | F | 47880 | 47910 | 0 | 2.30E-55 | IGS | LSC |
| R24 | 113 | F | 47880 | 47904 | 0 | 5.63E-59 | IGS | LSC |
| R25 | 119 | F | 47880 | 47898 | 0 | 1.37E-62 | IGS | LSC |
| R26 | 125 | F | 47880 | 47892 | 0 | 3.35E-66 | IGS | LSC |
| R27 | 131 | F | 47880 | 47886 | 0 | 8.19E-70 | IGS | LSC |
| R28 | 35 | F | 47880 | 47982 | 0 | 5.14E-12 | IGS | LSC |
| R29 | 41 | F | 47880 | 47976 | 0 | 1.25E-15 | IGS | LSC |
| R30 | 47 | F | 47880 | 47970 | 0 | 3.06E-19 | IGS | LSC |
| R31 | 53 | F | 47880 | 47964 | 0 | 7.48E-23 | IGS | LSC |
| R32 | 59 | F | 47880 | 47958 | 0 | 1.83E-26 | IGS | LSC |
| R33 | 65 | F | 47880 | 47952 | 0 | 4.46E-30 | IGS | LSC |
| R34 | 71 | F | 47880 | 47946 | 0 | 1.09E-33 | IGS | LSC |
| R35 | 77 | F | 47880 | 47940 | 0 | 2.66E-37 | IGS | LSC |
| R36 | 83 | F | 47880 | 47934 | 0 | 6.49E-41 | IGS | LSC |
| R37 | 89 | F | 47880 | 47928 | 0 | 1.58E-44 | IGS | LSC |
| R38 | 95 | F | 47880 | 47922 | 0 | 3.87E-48 | IGS | LSC |
| R39 | 40 | F | 59978 | 59997 | 1 | 2.39E-12 | IGS | LSC |
| R40 | 31 | P | 66505 | 66551 | 0 | 1.32E-09 | IGS | LSC |
| R41 | 38 | P | 76950 | 76955 | 3 | 5.96E-07 | IGS | LSC |
| R42 | 54 | F | 93971 | 94037 | 3 | 4.08E-16 | ycf2、ycf2 | LSC |
| R43 | 39 | F | 93972 | 93996 | 1 | 9.32E-12 | ycf2、ycf2 | LSC |
| R44 | 57 | F | 93972 | 94062 | 2 | 5.37E-20 | ycf2、ycf2 | LSC |
| R45 | 31 | F | 93974 | 94088 | 1 | 4.86E-07 | ycf2、ycf2 | LSC |
| R46 | 42 | F | 93983 | 94025 | 3 | 3.17E-09 | ycf2、ycf2 | LSC |
| R47 | 46 | F | 93983 | 94073 | 1 | 6.71E-16 | ycf2、ycf2 | LSC |
| R48 | 52 | F | 93983 | 94049 | 3 | 5.82E-15 | ycf2、ycf2 | LSC |
| R49 | 31 | F | 93998 | 94088 | 0 | 1.32E-09 | ycf2、ycf2 | LSC |
| R50 | 31 | F | 94007 | 94049 | 3 | 5.21E-03 | ycf2、ycf2 | LSC |
| R51 | 31 | F | 94007 | 94073 | 3 | 5.21E-03 | ycf2、ycf2 | LSC |
| R52 | 35 | F | 94032 | 94080 | 3 | 2.96E-05 | ycf2、ycf2 | LSC |
| R53 | 55 | F | 94032 | 94056 | 3 | 1.08E-16 | ycf2、ycf2 | LSC |
| R54 | 31 | F | 94064 | 94088 | 3 | 5.21E-03 | ycf2、ycf2 | LSC |
| R55 | 30 | F | 95386 | 95446 | 1 | 1.88E-06 | ycf2、ycf2 | LSC |
| R56 | 42 | F | 95389 | 95434 | 0 | 3.14E-16 | ycf2、ycf2 | LSC |
| R57 | 57 | F | 95389 | 95419 | 0 | 2.92E-25 | ycf2、ycf2 | LSC |
| R58 | 72 | F | 95389 | 95404 | 0 | 2.72E-34 | ycf2、ycf2 | LSC |
| R59 | 30 | C | 101016 | 142517 | 2 | 2.64E-04 | IGS | IRa、IRb |
| R60 | 30 | R | 101016 | 101016 | 2 | 2.64E-04 | IGS | IRa |
| R61 | 31 | F | 104495 | 139034 | 3 | 5.21E-03 | IGS | IRa、IRb |
| R62 | 31 | P | 104495 | 104499 | 3 | 5.21E-03 | IGS | IRa |
| R63 | 31 | P | 115020 | 115025 | 3 | 5.21E-03 | IGS | SSC |
| R64 | 41 | P | 126287 | 126288 | 1 | 1.57E-13 | IGS | SSC |
| R65 | 40 | P | 126288 | 126288 | 0 | 5.02E-15 | IGS | SSC |
| R66 | 35 | P | 128580 | 128580 | 3 | 2.96E-05 | ycf1、ycf1 | SSC |
| R67 | 30 | R | 142517 | 142517 | 2 | 2.64E-04 | IGS | IRb |

F: Forward, R: Reverse, C: Complement, P: Palindromic.

**Table S4: Long repeat sequences in *S. sphenanthera* chloroplast genome**

| ID | Repeat Start 1 | Type | Size (bp) | Repeat Start 2 | Mismatch (bp) | E-value | Gene | Region |
| --- | --- | --- | --- | --- | --- | --- | --- | --- |
| R1 | 4700 | P | 31 | 31845 | 1 | 4.76E-07 | IGS | LSC |
| R2 | 6991 | P | 50 | 6991 | 0 | 4.69E-21 | IGS | LSC |
| R3 | 7149 | P | 31 | 7149 | 3 | 5.10E-03 | IGS | LSC |
| R4 | 8577 | P | 51 | 8577 | 3 | 2.15E-14 | IGS | LSC |
| R5 | 8681 | P | 35 | 46055 | 3 | 2.90E-05 | trnS-GCU | LSC |
| R6 | 8688 | F | 31 | 36413 | 3 | 5.10E-03 | trnS-GCU、trnS-UGA | LSC |
| R7 | 10323 | F | 31 | 37347 | 3 | 5.10E-03 | trnG-GCC、trnG-UCC | LSC |
| R8 | 13558 | F | 32 | 99141 | 1 | 1.23E-07 | IGS | LSC、IRa |
| R9 | 13558 | P | 32 | 140515 | 1 | 1.23E-07 | IGS | LSC、IRb |
| R10 | 14418 | P | 36 | 14418 | 0 | 1.26E-12 | IGS | LSC |
| R11 | 28375 | P | 31 | 28410 | 1 | 4.76E-07 | IGS | LSC |
| R12 | 30322 | F | 36 | 143069 | 0 | 1.26E-12 | ndhB-D2 | LSC、IRb |
| R13 | 30322 | P | 36 | 96583 | 0 | 1.26E-12 | ndhB | LSC、IRa |
| R14 | 36411 | P | 30 | 46055 | 3 | 1.84E-02 | trnS-UGA、trnS-GCU | LSC |
| R15 | 39557 | F | 30 | 41781 | 3 | 1.84E-02 | psaB、psaA | LSC |
| R16 | 39567 | F | 31 | 41791 | 3 | 5.10E-03 | psaB、psaA | LSC |
| R17 | 44491 | F | 41 | 98744 | 0 | 1.23E-15 | IGS | LSC、IRa |
| R18 | 44491 | P | 41 | 140903 | 0 | 1.23E-15 | IGS | LSC、IRb |
| R19 | 47522 | P | 44 | 47522 | 0 | 1.92E-17 | IGS | LSC |
| R20 | 47564 | P | 34 | 47570 | 2 | 1.30E-06 | IGS | LSC |
| R21 | 47568 | P | 30 | 47570 | 2 | 1.84E-05 | IGS | LSC |
| R22 | 58152 | F | 40 | 58171 | 1 | 2.34E-12 | IGS | LSC |
| R23 | 64629 | P | 31 | 64675 | 0 | 1.29E-09 | IGS | LSC |
| R24 | 92161 | F | 31 | 92257 | 0 | 1.29E-09 | ycf2 | LSC |
| R25 | 92161 | F | 32 | 92209 | 3 | 1.41E-03 | ycf2 | LSC |
| R26 | 92161 | F | 38 | 92233 | 3 | 5.84E-07 | ycf2 | LSC |
| R27 | 92176 | F | 30 | 92224 | 3 | 1.84E-02 | ycf2 | LSC |
| R28 | 92176 | F | 54 | 92200 | 3 | 4.00E-16 | ycf2 | LSC |
| R29 | 92189 | F | 65 | 92213 | 3 | 1.68E-22 | ycf2 | LSC |
| R30 | 92200 | F | 36 | 92248 | 3 | 7.91E-06 | ycf2 | LSC |
| R31 | 92218 | F | 42 | 92242 | 3 | 3.11E-09 | ycf2 | LSC |
| R32 | 99128 | C | 30 | 140530 | 2 | 2.58E-04 | IGS | IRa、IRb |
| R33 | 99128 | R | 30 | 99128 | 2 | 2.58E-04 | IGS | IRa |
| R34 | 99757 | C | 30 | 139902 | 3 | 1.84E-02 | IGS | IRa、IRb |
| R35 | 102596 | F | 31 | 137058 | 3 | 5.10E-03 | IGS | IRa、IRb |
| R36 | 102596 | P | 31 | 102600 | 3 | 5.10E-03 | IGS | IRa |
| R37 | 110733 | P | 91 | 128864 | 2 | 4.58E-40 | ndhF、ycf1 | IRa、IRb |
| R38 | 110733 | P | 91 | 128864 | 1 | 1.05E-42 | ndhF、ycf1 | IRa、IRb |
| R39 | 110734 | P | 90 | 128864 | 1 | 1.05E-42 | ndhF、ycf1 | IRa、IRb |
| R40 | 110735 | P | 89 | 128864 | 2 | 4.58E-40 | ndhF、ycf1 | IRa、IRb |
| R41 | 115677 | P | 30 | 115677 | 2 | 2.58E-04 | ccsA | SSC |
| R42 | 124453 | P | 32 | 124495 | 0 | 3.22E-10 | IGS | SSC |
| R43 | 126775 | P | 51 | 126775 | 3 | 2.15E-14 | ycf1 | SSC |
| R44 | 140530 | R | 30 | 140530 | 2 | 2.58E-04 | IGS | IRb |

F: Forward, R: Reverse, C: Complement, P: Palindromic.

**Table S5: Long repeat sequences in *K. coccinea* chloroplast genome**

| ID | Repeat Start 1 | Type | Size (bp) | Repeat Start 2 | Mismatch (bp) | E-value | Gene | Region |
| --- | --- | --- | --- | --- | --- | --- | --- | --- |
| R1 | 4700 | P | 31 | 31845 | 1 | 4.76E-07 | IGS | LSC |
| R2 | 6991 | P | 50 | 6991 | 0 | 4.69E-21 | IGS | LSC |
| R3 | 7149 | P | 31 | 7149 | 3 | 5.10E-03 | IGS | LSC |
| R4 | 8577 | P | 51 | 8577 | 3 | 2.15E-14 | IGS | LSC |
| R5 | 8681 | P | 35 | 46055 | 3 | 2.90E-05 | trnS-GCU | LSC |
| R6 | 8688 | F | 31 | 36413 | 3 | 5.10E-03 | trnS-GCU、trnS-UGA | LSC |
| R7 | 10323 | F | 31 | 37347 | 3 | 5.10E-03 | trnG-GCC、trnG-UCC | LSC |
| R8 | 13558 | F | 32 | 99141 | 1 | 1.23E-07 | IGS | LSC、IRa |
| R9 | 13558 | P | 32 | 140515 | 1 | 1.23E-07 | IGS | LSC、IRb |
| R10 | 14418 | P | 36 | 14418 | 0 | 1.26E-12 | IGS | LSC |
| R11 | 28375 | P | 31 | 28410 | 1 | 4.76E-07 | IGS | LSC |
| R12 | 30322 | F | 36 | 143069 | 0 | 1.26E-12 | ndhB-D2 | LSC、IRb |
| R13 | 30322 | P | 36 | 96583 | 0 | 1.26E-12 | ndhB | LSC、IRa |
| R14 | 36411 | P | 30 | 46055 | 3 | 1.84E-02 | trnS-UGA、trnS-GCU | LSC |
| R15 | 39557 | F | 30 | 41781 | 3 | 1.84E-02 | psaB、psaA | LSC |
| R16 | 39567 | F | 31 | 41791 | 3 | 5.10E-03 | psaB、psaA | LSC |
| R17 | 44491 | F | 41 | 98744 | 0 | 1.23E-15 | IGS | LSC、IRa |
| R18 | 44491 | P | 41 | 140903 | 0 | 1.23E-15 | IGS | LSC、IRb |
| R19 | 47522 | P | 44 | 47522 | 0 | 1.92E-17 | IGS | LSC |
| R20 | 47564 | P | 34 | 47570 | 2 | 1.30E-06 | IGS | LSC |
| R21 | 47568 | P | 30 | 47570 | 2 | 1.84E-05 | IGS | LSC |
| R22 | 58152 | F | 40 | 58171 | 1 | 2.34E-12 | IGS | LSC |
| R23 | 64629 | P | 31 | 64675 | 0 | 1.29E-09 | IGS | LSC |
| R24 | 92161 | F | 31 | 92257 | 0 | 1.29E-09 | ycf2 | LSC |
| R25 | 92161 | F | 32 | 92209 | 3 | 1.41E-03 | ycf2 | LSC |
| R26 | 92161 | F | 38 | 92233 | 3 | 5.84E-07 | ycf2 | LSC |
| R27 | 92176 | F | 30 | 92224 | 3 | 1.84E-02 | ycf2 | LSC |
| R28 | 92176 | F | 54 | 92200 | 3 | 4.00E-16 | ycf2 | LSC |
| R29 | 92189 | F | 65 | 92213 | 3 | 1.68E-22 | ycf2 | LSC |
| R30 | 92200 | F | 36 | 92248 | 3 | 7.91E-06 | ycf2 | LSC |
| R31 | 92218 | F | 42 | 92242 | 3 | 3.11E-09 | ycf2 | LSC |
| R32 | 99128 | C | 30 | 140530 | 2 | 2.58E-04 | IGS | IRa、IRb |
| R33 | 99128 | R | 30 | 99128 | 2 | 2.58E-04 | IGS | IRa |
| R34 | 99757 | C | 30 | 139902 | 3 | 1.84E-02 | IGS | IRa、IRb |
| R35 | 102596 | F | 31 | 137058 | 3 | 5.10E-03 | IGS | IRa、IRb |
| R36 | 102596 | P | 31 | 102600 | 3 | 5.10E-03 | IGS | IRa |
| R37 | 110733 | P | 91 | 128864 | 2 | 4.58E-40 | ndhF、ycf1 | IRa、IRb |
| R38 | 110733 | P | 91 | 128864 | 1 | 1.05E-42 | ndhF、ycf1 | IRa、IRb |
| R39 | 110734 | P | 90 | 128864 | 1 | 1.05E-42 | ndhF、ycf1 | IRa、IRb |
| R40 | 110735 | P | 89 | 128864 | 2 | 4.58E-40 | ndhF、ycf1 | IRa、IRb |
| R41 | 115677 | P | 30 | 115677 | 2 | 2.58E-04 | ccsA | SSC |
| R42 | 124453 | P | 32 | 124495 | 0 | 3.22E-10 | IGS | SSC |
| R43 | 126775 | P | 51 | 126775 | 3 | 2.15E-14 | ycf1 | SSC |
| R44 | 140530 | R | 30 | 140530 | 2 | 2.58E-04 | IGS | IRb |

F: Forward, R: Reverse, C: Complement, P: Palindromic.
